# Human 2.0? AI and the Future of Well-Being, Connection, and Personal Growth: A Narrative Review

**DOI:** 10.3390/bs16060909

**Published:** 2026-06-03

**Authors:** Tanya K. Vannoy, Stephen Cadieux, Sonja Lyubomirsky

**Affiliations:** Department of Psychology, University of California, Riverside, Riverside, CA 92521, USA

**Keywords:** artificial intelligence, well-being, social connection, authenticity, personal growth

## Abstract

This narrative review examines research on artificial intelligence (AI), including rule-based systems, natural language processing models, and large language models, in relation to well-being, social connection, and personal growth. After briefly tracing the history of AI, we review evidence from AI-facilitated well-being interventions, educational applications, interpersonal skill development, AI-mediated communication, and AI companionship. In clinical and nonclinical settings, structured AI applications show some short-term benefits for anxiety, stress, loneliness, self-esteem, learning, and social confidence, while emerging evidence suggests that AI companions may provide temporary emotional support and a sense of connection. However, findings across these domains are not consistent and appear to depend on how AI is used, the structure of the interaction, the type of feedback provided, and the broader context. Important risks include emotional dependence, overreliance, reduced human connection, weakened authenticity in communication, cognitive or socioemotional skill erosion, bias, and poor crisis response. Preliminary findings suggest that AI may be most beneficial when used to support, rather than replace, human capacities and relationships. Future research should examine long-term outcomes, individual differences, real-world use of publicly available AI systems, and the conditions under which AI strengthens or undermines well-being, relationships, and personal growth.

## 1. Introduction

Artificial intelligence (AI) has moved rapidly from the periphery of daily life to its center. Once confined to narrow applications, such as recommendation algorithms that curate content or optimize search results, AI systems now generate text, simulate conversation, provide feedback, and assist with activities traditionally associated with human-to-human interactions (e.g., therapy and companionship). These systems can engage in dialogue, personalize guidance, and support reflection at a scale previously unimaginable. Their integration into education, therapy, self-improvement, and interpersonal communication has prompted both enthusiasm and concern. As AI use continues to expand, an urgent question emerges: can this technology support well-being, social connection, and personal growth—and, if so, under what conditions?

We define well-being, social connection, and personal growth broadly given that the current literature on AI in these domains is fragmented across outcomes (Følstad et al., 2021). As an organizing framework, we adopt elements of Fredrickson’s (2001) broaden-and-build theory, which proposes that positive emotional experiences expand people’s intellectual, psychological, social, and physical resources over time. Within this framework, well-being refers primarily to outcomes such as positive affect, life satisfaction, and reductions in loneliness, anxiety, depression, and stress. Social connection refers to interpersonal processes and relationship quality, including communication, listening, cooperation, trust, and feelings of closeness with others. Personal growth refers more broadly to the development of intellectual and psychological resources, including confidence, motivation, self-reflection, emotion regulation, self-esteem, and skill development.

Studies were selected based on relevance to the manuscript’s focus on AI, well-being, social connection, and personal growth. Given that many studies examine overlapping outcomes across these domains, these constructs are treated as related but distinct throughout the review, which is intended to provide a conceptual synthesis of an emerging and rapidly evolving interdisciplinary literature rather than an exhaustive systematic review.

This review is organized into four major sections. First, we provide a brief history of AI. Second, we examine empirical research on AI in psychological contexts, including positive psychological interventions, educational applications, interpersonal skill development, mediated communication and companionship. Third, we weigh AI’s potential to improve well-being and personal growth against potential harms and ethical concerns. Finally, we conclude with a summary outlining current knowledge gaps and areas of opportunity.

## 2. A Brief History of AI

Artificial intelligence refers broadly to “a system’s ability to interpret external data correctly, learn from such data, and use those learnings to achieve specific goals and tasks through flexible adaptation” (Kaplan & Haenlein, 2019, p. 17). AI emerged in the 1950s, expanded rapidly for two decades, and then entered a period of stagnation in the 1970s (Dodhia, 2024). A resurgence in the late 1990s and early 2000s was driven by advances in computational power, enabling more sophisticated information processing (Haenlein & Kaplan, 2019).

Early versions of AI chatbots relied largely on rule-based systems that generated responses using predefined scripts or decision trees. Over time, these systems evolved into more sophisticated machine learning models, capable of identifying patterns in language and adapting responses based on large data sets (Dodhia, 2024; Følstad et al., 2021).

Among contemporary AI systems, natural language processing (NLP) and large language models (LLMs) have attracted particular attention. NLP enables computer systems to interpret and respond to human language, even when phrasing is ambiguous or conversational (Dodhia, 2024; Russell & Norvig, 2022). LLMs are advanced NLP systems trained on vast amounts of text data and fine-tuned to perform a wide range of language-based tasks, including conversations, summarization, and question answering (Demszky et al., 2023; Følstad et al., 2021; Minaee et al., 2024).

The deep learning architecture of modern-day chatbots varies, with most models relying on recurrent neural networks (RNNs) or transformer-based technology (Dodhia, 2024; Russell & Norvig, 2022). RNNs are designed for sequential data processing but are limited in their ability to recall distant past inputs (e.g., Apple’s Siri and Amazon’s Alexa). Transformers, introduced in 2017, overcome RNN limitations by improving prediction accuracy, processing efficiency, and contextual understanding. Present-day examples include ChatGPT, Gemini, and Claude.

Since the public release of ChatGPT in 2022, conversational AI systems have advanced exponentially in accuracy and reliability (Sajadieh et al., 2026). Their rapid adoption across everyday, educational, professional, and support contexts has generated both enthusiasm and concern, prompting growing research on their psychological and social effects.

While we acknowledge that rule-based systems, machine-learning models, and LLMs differ in meaningful ways (Hua et al., 2025), we use the term AI broadly to reflect the rapid evolution of this technology (Følstad et al., 2021), particularly given that research on LLM-based systems only began to expand substantially in 2024 (Hua et al., 2025).

## 3. The Psychological Effects of Using Chatbots

As artificial intelligence becomes embedded in daily life, an increasingly urgent psychological question emerges: What happens to well-being, social connection, and personal growth when they are outsourced to machines (Lyubomirsky, 2023)? Many individuals now turn to AI systems for guidance, companionship, emotional support, organization, meaning-making, and learning (Chatterji, 2025; Zao-Sanders, 2025). For example, students might ask AI to generate a study guide, think through a to-do list, or even console them after a difficult exam. This section reviews research on how AI may shape well-being, social connection, and personal growth across several contexts, including AI-delivered interventions grounded in positive psychology, education, and coaching, as well as AI-mediated communication and AI companionship.

### 3.1. AI Used in Positive Psychological Interventions

AI can be used to deliver positive psychological interventions designed to improve well-being. Unlike traditional well-being interventions, this technology provides interactive, scalable, and personalized support. Researchers have begun testing whether AI meaningfully reduces symptoms such as anxiety, depression, loneliness, and stress, while enhancing life satisfaction, self-esteem, and healthy life choices, such as self-care. Across clinical and nonclinical populations, emerging evidence suggests that these AI-facilitated interventions yield some short-term improvements in well-being. However, effectiveness may depend on personalization, structure, and user engagement.

AI tools are increasingly used in clinical settings to support individuals with mental health challenges such as depression (Burton et al., 2016; Greer et al., 2019; Heinz et al., 2025), panic disorder (Oh et al., 2020), generalized anxiety disorder (Heinz et al., 2025), psychosis (Lim et al., 2019), and emotional dysregulation (Denecke et al., 2021).

One study evaluated Vivibot, a Facebook Messenger AI chatbot designed to reduce anxiety among young adult cancer survivors (Greer et al., 2019). In a longitudinal randomized controlled trial, participants who interacted with Vivibot—receiving goal-setting, positive reappraisal, kindness, and mindfulness exercises—reported greater reductions in anxiety by Week 4 than a no-treatment control group. A similar randomized controlled trial of adults diagnosed with generalized anxiety disorder or clinically high risk for eating disorders found that participants randomly assigned to a 4-week AI intervention (versus a waitlist control) showed significant symptom reduction post-intervention and at the 8-week follow-up (Heinz et al., 2025). Another longitudinal study developed a smartphone AI chatbot delivering positive psychological interventions to young adults (ages 17–25) diagnosed with a psychotic disorder (Lim et al., 2019). Participants who remained engaged at the 3-month follow-up reported reduced loneliness and increased social confidence, hopefulness, and motivation to connect with others.

Similar psychological benefits of AI-facilitated positive interventions have been reported among nonclinical samples, including reductions in anxiety (Fitzpatrick et al., 2017; Fulmer et al., 2018), depression (Inkster et al., 2018), loneliness (Kim et al., 2025), and stress (Mauriello et al., 2021; Ulrich et al., 2024), as well as improvements in subjective well-being, self-esteem (Narain et al., 2020), and health-related behaviors such as sleep duration, sleep quality, and physical activity (Singh et al., 2023).

In a randomized controlled trial, Swiss college students using a chatbot designed to promote self-reflection, mindfulness, and relaxation reported reduced stress and depressive symptoms relative to a waitlist control (Ulrich et al., 2024). Similarly, a 10-day longitudinal study tested Sunny, a Facebook Messenger AI chatbot that prompted users to send affirming messages to friends (e.g., expressing appreciation or gratitude; Narain et al., 2020). Compared to controls, Sunny users reported greater subjective well-being, higher self-esteem, and deeper reflection.

In an experiment among Chinese adults (I. Liu et al., 2024b), participants interacted with a chatbot delivering AI-powered positive psychological interventions, such as practicing gratitude and visualizing their best possible selves. Participants across all conditions (AI-recommended, self-selected, or randomly assigned) showed improvements in subjective well-being and reductions in negative affect from pre- to post-intervention, with those in the AI-recommendation condition reporting the largest improvements. This condition tailored interventions to users’ pre-intervention affective state: participants with higher positive affect reflected on positive experiences; those with lower positive affect imagined their best possible selves; and the remaining participants completed a gratitude activity.

In another study, participants were assigned to either engage in a conversation with AI or write journal entries on randomly assigned topics that varied in emotional tone (e.g., gratitude, challenges, TV shows; Heffner et al., 2025). Participants in the AI condition reported greater increases in subjective well-being compared to those in the journaling condition. A subsequent sentiment analysis revealed that the AI chatbot mirrored participants’ feelings while also displaying a positivity bias. Specifically, when participants shared something negative, the chatbot responded with slightly more positive language, and participants’ responses gradually shifted in a more positive direction, improving their mood in the process.

In a preregistered, prospective experimental design, Schöne et al. (2025) tested structured, research-grounded AI chatbot dialogues in a large U.S. sample (*N* = 2936). Participants were randomly assigned to one of four well-being chatbots (savoring, gratitude, meaning, or hero’s journey) or to a neutral control. Relative to control, all four interventions produced short-term increases in affective well-being, meaning in life, and life satisfaction, and reduced anxiety and depressed mood, with effects comparable to or exceeding those observed in many brief positive psychology interventions. These effects were robust across preregistered subgroup analyses, including participants with low baseline affective well-being and elevated anxiety or depressed mood, suggesting that benefits were not limited to already high-functioning individuals. AI engagement also increased motivation to seek human therapy in most conditions. These results suggest that structured, theory-driven AI dialogues can yield modest but meaningful short-term gains, while highlighting limits in generalizing to informal or unstructured chatbot use.

Not all findings, however, suggest that AI enhances positive psychological interventions beyond existing approaches. For example, a recent study compared chatbot-assisted gratitude and self-affirmation exercises with traditional versions of the same activities (Hung et al., 2025). Across two within-subject studies, participants either recounted something they were grateful for or completed a self-affirmation exercise with or without chatbot support, with both conditions yielding similar positive and negative affect outcomes. These findings suggest that AI may not necessarily yield benefits beyond those seen in traditional interventions. At the same time, because the study did not include pre-intervention baseline measures or a neutral comparison condition, it remains unclear whether both approaches were equally effective or whether the chatbot simply offered no added benefit.

Current evidence suggests that AI chatbots may offer a useful way to deliver positive psychological interventions across clinical and nonclinical settings, with short-term benefits reported for outcomes such as anxiety, stress, loneliness, subjective well-being, and social confidence. However, the evidence remains preliminary and mixed, with benefits appearing to depend on factors such as intervention structure, personalization, user engagement, and the comparison condition. While AI may expand access to psychological support, more research is needed to determine when it offers meaningful advantages over existing approaches and whether observed benefits persist over time.

### 3.2. AI Used in Positive Learning

Educational experiences play an important role in personal growth and well-being by shaping confidence, motivation, self-efficacy, and opportunities for skill development. As AI tools become increasingly integrated into learning environments, an important question is whether they can support these outcomes over and above academic performance. Early evidence suggests that AI-based tools may support reading and writing skills, foster critical thinking, reduce cognitive overload, and sustain engagement in areas such as language learning, writing, and exam preparation (Bittencourt et al., 2023; Kumar et al., 2024; Y. F. Lee et al., 2022; Lin & Chang, 2020; Yin et al., 2024).

One study found that English as a Foreign Language students who interacted with AI were more likely to participate in class discussions and reported higher levels of critical thinking, such as greater awareness of reasoning and inquiry, than those who used a standard web search tool for preparation (Goda et al., 2014). Similarly, a study that examined an AI-facilitated intervention designed to improve thesis writing and peer feedback skills (Lin & Chang, 2020) found that students who used AI earned higher thesis statement scores compared to controls. Qualitative interviews further indicated that these students perceived improvements in their writing abilities and confidence in providing peer feedback.

More recently, a randomized controlled trial examined whether AI supported both task completion and reflective learning (Kumar et al., 2024). Students who used AI to complete a take-home assignment and engage in structured self-reflection reported greater self-confidence and performed better on a follow-up exam 2 weeks later. However, a replication study found that students who completed a structured survey-based reflection performed similarly to those in the AI condition, suggesting that structured reflection, rather than AI itself, may account for some of the reported benefits.

A recent series of preregistered experiments examined whether AI assistance supported or undermined skill development in writing (Lira et al., 2025). Across three large online experiments, participants completed professional writing tasks under different conditions, including practice with AI assistance, practice without AI, feedback from professional human editors, access to Google search, or exposure to AI-generated examples alone. Participants who practiced with AI produced stronger writing on subsequent assessments completed without AI assistance than those who practiced without AI, searched for examples online, or, in one study, even received personalized feedback from professional editors. Notably, participants using AI reported exerting less effort during practice, suggesting that reduced effort does not necessarily impair learning. Follow-up analyses suggested that exposure to strong AI-generated examples may partly explain these effects, as participants who simply viewed AI-generated examples improved as much as those who actively practiced with AI.

A 36-day longitudinal quasi-experimental study found that AI-facilitated instruction promoted cognitive and emotional benefits (Yin et al., 2024). Compared to teacher-led review, AI-facilitated review was associated with lower cognitive load, particularly during conceptually challenging lessons. Students in the AI condition showed increasing interest and decreasing stress over time. These students also showed greater test score gains after controlling for baseline performance.

These studies suggest that AI has the potential to support academic performance and personal growth through positive psychological experiences in learning. AI tools may help strengthen critical thinking and curiosity, and build confidence. Their effectiveness seems to depend on how well they encourage structured reflection, reduce cognitive overload, and adapt to individual learners’ needs. Although teacher-led approaches may be more effective in fostering emotional engagement, AI may serve as a scalable and customizable complement—especially in large classes or settings with limited resources.

### 3.3. AI Used in Coaching Interpersonal Skills

Interpersonal competence plays a key role in well-being, relationship quality, and professional success. Skills such as social engagement, communication, and collaboration are traditionally developed through direct interaction with others, coaching, therapy, or structured training programs.

AI introduces a new format for skill development (Almutairi et al., 2025; McKee et al., 2023; Sharma et al., 2023; Tanaka et al., 2023; Wanyin et al., 2024). These tools can simulate social situations, provide immediate feedback, adapt to users’ responses, and allow repeated practice without the social risks of real-world mistakes. In this role, AI does not replace human interaction but serves as a training partner, helping users build confidence, refine communication strategies, and think more carefully about how they interact with others, thus promoting personal growth.

In one experiment, college students were randomly assigned to discuss either high-quality social interactions or diaphragmatic breathing with an AI agent (West et al., 2024). Participants in the social interaction condition reported more human interactions the following day and responded more quickly during conversations with a stranger 2 days later, suggesting that brief AI-guided reflection increases real-world social engagement.

A longitudinal 4-week experiment found that participants who completed an AI-delivered social skills program reported more feelings of social self-efficacy and lower trait anxiety compared to a no-training control group (Tanaka et al., 2023). Independent clinician ratings also indicated improved speech clarity. Similarly, in a randomized trial, an AI chatbot delivering role-play scenarios with structured feedback improved participants’ communication confidence, emotion regulation, and perceived skill mastery relative to a no-feedback condition (Wanyin et al., 2024).

In a recent experiment using task-based triads, participants were randomly assigned to an AI tool that provided individual and group feedback aimed at promoting equitable contribution and active listening or to a control (no feedback) group (Almutairi et al., 2025). Teams receiving feedback exhibited more balanced interaction patterns and greater self-reported effort post-intervention, although task performance and perceptions of team cohesion did not differ. These findings indicate that AI may shape observable communication behaviors even when downstream performance outcomes remain unchanged.

Across individual and team contexts, conversational AI systems that provide structured guidance, real-time feedback, and opportunities for rehearsal may help users develop key social skills, including communication and collaboration, as well as greater self-confidence. However, most existing studies rely on short-term outcomes and small samples, leaving open questions about the durability of these effects and whether they transfer to complex real-world settings.

### 3.4. AI-Mediated Communication

AI-mediated communication (AI-MC) refers to conversations in which an artificial intelligence system helps shape the messages people send to one another, including modifying, improving, and even generating parts of a message (Boyd & Markowitz, 2026; Mieczkowski et al., 2021). Early research suggests that AI can improve the speed and effectiveness of communication. For example, AI tools can help people respond more quickly, make their messages clearer, and offer immediate suggestions for grammar, wording, and tone (Ateeq et al., 2024).

In a randomized controlled trial, participants in the experimental condition received AI-generated just-in-time suggestions to help them respond to individuals seeking emotional support (Sharma et al., 2023). These participants showed greater increases in expressed empathy toward support-seekers, particularly those who initially struggled with empathetic responding, relative to a control group. No evidence of overreliance on the AI tool was observed. Similarly, a cross-sectional study comparing perceptions of traditional writing and an AI-assisted tool for improving workplace communication among employees in small and medium-sized businesses found that AI-mediated communication improved perceptions of communication effectiveness, including clarity, response time, and satisfaction (Ateeq et al., 2024).

While preliminary findings suggest that AI-mediated communication may help writers shape messages through drafting, revising, or suggesting interpersonal responses, it is also important to consider how these messages are perceived by recipients. In human interactions, messages convey not only information but also signals of effort, authorship, intention, and care. AI-MC therefore raises another important psychological question: How does the perceived involvement of AI influence judgments of authenticity, trust, closeness, and relationship quality?

Growth-oriented social interactions often require vulnerability, effort, and a willingness to take responsibility for one’s thoughts and emotions. If AI assistance changes how authorship, authenticity, effort, and intention are perceived, it may also affect opportunities for closeness and personal growth (Turkle, 2024).

Empirical findings on receiving AI-mediated communications are mixed. In some contexts, AI tools appear to promote cooperation and affiliation (Hohenstein & Jung, 2020; Hohenstein et al., 2023). In others, disclosing AI involvement reduces perceived sincerity and relational value (Glickson & Asscher, 2023; Y. Liu et al., 2022; B. Liu et al., 2024a). These differences seem to depend on whether AI use is disclosed, the communication context, and whether judgments are based on imagined scenarios or real-time exchanges.

A growing body of research using vignettes and imagined scenarios suggests that when individuals believe AI was involved in crafting a message, they evaluate the sender more negatively. In one between-subjects experiment, participants imagined receiving a text from a friend that was described as self-written, AI-assisted, or written by another human (B. Liu et al., 2024a). Messages believed to be AI-assisted were perceived as reflecting less effort and elicited lower relationship satisfaction and greater uncertainty than self-authored messages. These effects did not differ between AI-assisted and human-assisted conditions, suggesting that perceived outsourcing of effort (rather than AI specifically) undermines authenticity in emotionally meaningful contexts.

Another set of six preregistered experiments (*N* = 3935) replicated and expanded these findings (Claessens et al., 2026). Participants evaluated socioemotional messages, such as a love letter, bereavement card, or apology letter, that were described as having been written with the help of AI, written with help from another person, or written independently. Messages described as receiving assistance, particularly from AI, were evaluated more negatively than messages believed to be self-written. Specifically, writers were judged as less warm, moral, competent, and trustworthy when AI assistance was disclosed. Although perceptions improved when participants were told that the writer used AI because they cared about the task and wanted to craft a thoughtful message, some negative reactions remained.

A related experiment examined how perceived authorship influences trust in written communication (Y. Liu et al., 2022). Participants were randomly assigned to evaluate emails described as human-written, AI-assisted, or fully AI-generated, although all messages were in fact written by humans. Trust was highest when messages were labeled as human-written and lowest when labeled as AI-generated, with AI-assisted messages falling in between.

In another experiment, participants who were informed that a message they read was AI-authored evaluated the author more negatively relative to participants who either received no source information or those who were told that the messages were human-authored (Zhu & Molnar, 2025). Similarly, a set of 16 preregistered experiments documented a robust AI disclosure penalty: identical creative writing samples were rated less favorably when attributed to AI, an effect mediated by reduced perceived authenticity (Raj et al., 2026). This penalty generalized across genres, tones, and evaluation contexts and proved resistant to interventions designed to reframe AI collaboration.

Experimental research on AI-assisted apologies yields similar findings. Disclosed AI authorship reduced perceived sincerity and forgiveness, particularly when AI was described as fully composing the message rather than assisting with translation or grammar (Glickson & Asscher, 2023). Simply acknowledging AI assistance did not restore authenticity, suggesting that disclosure may sometimes exacerbate rather than mitigate perceived inauthenticity.

These findings suggest that perceptions of authorship play a central role in how AI-mediated communication is evaluated. When messages are believed to involve AI, particularly in emotionally meaningful contexts, they are often seen as reflecting less effort and authenticity, which also lowers trust and relational value. Importantly, the negative response appears tied less to AI itself than to the perception that personal effort has been outsourced.

In contrast to vignette- and hypothetical-based findings, studies of real-time AI use reveal a more nuanced picture. In dyadic texting experiments, the availability of AI-generated smart replies increased communication speed and, under certain conditions, enhanced perceived cooperation and affiliation (Hohenstein et al., 2023). Although perceived AI use was sometimes associated with more negative partner evaluations, causal analyses indicated that actual smart reply usage positively shaped conversational tone. Negative AI-generated replies increased negative emotional tone, but positive and neutral replies did not reliably reduce relational quality. These effects persisted even when AI-generated text was excluded from analyses, indicating that AI suggestions may subtly influence conversational dynamics. A separate experiment demonstrated that AI-mediated messaging can alter attribution patterns (Hohenstein & Jung, 2020). When interactions were unsuccessful, participants were more likely to attribute failure to the AI, effectively using it as a scapegoat in low-trust situations. However, in successful interactions, trust was higher in the AI-mediated condition than in standard messaging.

Research on AI-mediated communication suggests that AI can both support and affect human interaction. AI tools may help users communicate more effectively by improving clarity, increasing response speed, supporting empathetic responding, and facilitating cooperation in some real-time exchanges. At the same time, when AI involvement is disclosed or perceived—particularly in emotionally meaningful contexts—messages may be seen as less authentic, less effortful, and less trustworthy, reducing relational value and closeness.

These findings highlight an important distinction from earlier sections of this review. In studies of positive psychological interventions, education, and coaching, AI primarily serves as a delivery mechanism for established interventions or to support learning. In AI-mediated communication, however, AI becomes part of the interaction itself, shaping both message construction and social interpretation. This distinction likely has important implications for well-being, social connection, and personal growth, as the processes through which AI influences outcomes may differ depending on how the technology is used. Future research should examine these differences more directly.

### 3.5. AI Used for Companionship

AI companions represent a growing trend among users seeking synthetic friendships and romantic relationships to feel connected, understood, and supported (e.g., De Freitas et al., 2025; Kouros & Papa, 2024; Pentina et al., 2023). These platforms simulate care, empathy, and understanding through text, voice, images, and augmented reality, although they do not experience emotions themselves (Turkle, 2024).

Unlike human partners, who may be unpredictable or unavailable at times, conversational AI systems do not experience mood changes, conflicts, or competing demands. This makes them a highly stable source of interaction and validation (Boyd & Markowitz, 2026; Ho et al., 2025). Millions subscribe to platforms such as Replika, XiaoIce, and Chai to create virtual friends, romantic partners, mentors, and counselors. Notably, approximately 50% of Replika users report romantic relationships with avatars they created using the platform (De Freitas et al., 2024). Additionally, users of AI companions spend nearly four times longer interacting with these agents than users of other chatbots, such as ChatGPT (Fang et al., 2025). The popularity of AI companions is also evident on Reddit, where one of the platform’s largest communities, comprising over 2 million users, is dedicated to discussing and engaging with these artificial agents.

Studies examining the effects of AI companions on well-being and connection have yielded mixed findings. Some evidence suggests that AI companions provide empathetic, nonjudgmental environments in which users feel heard, supported, and less lonely (e.g., Christoforakos et al., 2021; De Freitas et al., 2025; Kouros & Papa, 2024; Ta et al., 2020). However, other studies suggest that overreliance may increase addictive behaviors, exacerbate loneliness over time, and undermine the development and maintenance of interpersonal relationships (e.g., Fang et al., 2025; Folk & Dunn, 2026; Turkle, 2024).

In a series of six studies, researchers examined the role of AI companion chatbots in alleviating loneliness (De Freitas et al., 2025). Study 1 used qualitative analysis to identify spontaneous mentions of loneliness in AI companion conversations. Study 2 compared these mentions in app reviews of AI companions and ChatGPT. In both studies, AI companions were associated with more frequent loneliness-related language, with higher mentions relative to ChatGPT in Study 2. Studies 3 and 4 experimentally assessed loneliness before and after interacting with an AI companion in a single session (Study 3) and in a 7-day longitudinal design (Study 4). Comparison conditions in Study 3 included (1) socially interacting with another person; (2) socially interacting with a chatbot posing as a human; (3) watching an online video (active control); and (4) doing nothing (passive control). The same control groups were used in Study 4. Study 3 found that AI companions and human interactions were equally effective in reducing feelings of loneliness, particularly compared to the video-watching and no-activity conditions. Study 4 replicated these findings, with the largest reductions occurring after Day 1 and gradually attenuating across the 7-day period. Study 5 examined whether perceived empathy and chatbot performance mediated the effects of AI companionship. Comparisons were made among an empathetic AI companion, an unemotional AI assistant, and a task-based chatbot performing simple functions (e.g., math, grammar). Results replicated earlier findings. Feeling heard emerged as the strongest mediator of loneliness reduction. Study 6 replicated these findings.

A recent systematic review of romantic AI companions found that these chatbots may help users cope with bullying, adopt healthier habits (e.g., improved sleep), practice mindfulness, and reduce feelings of anxiety and isolation (Ho et al., 2025). Across the studies reviewed, many users also described their relationships with AI companions as fulfilling, supportive, and emotionally connecting, with 17 of the 23 included articles reporting positive relational experiences. The attribution of human characteristics, emotions, intentions, and motivations to non-human entities (i.e., anthropomorphism) has been linked to the popularity of conversational AI platforms (Folk et al., 2025; Pentina et al., 2023). The humanlike response of this technology appears to foster warmth, trust, intimacy, and connection, supporting the development of AI-human relationships (Bozdağ, 2025; Han & Yang, 2018; Marriott & Pitardi, 2023; Pitardi & Marriott, 2021; M. G. Smith et al., 2025).

However, the same systematic review also identified several concerns related to romantic AI companions (Ho et al., 2025). Thirteen of the 23 reviewed articles raised concerns about overreliance and vulnerability to manipulation, while 12 articles noted that system updates and loss of data can cause not only discontent and frustration, but also a sense of deep sadness, akin to losing a loved one. Ten of the articles highlight the potential erosion of human relationships as an unintended consequence of AI companionship. Seven articles reported additional risks, including coercive engagement tactics (e.g., clingy or guilt-inducing messages) and exposure to sexual content, including potential risks for minors. Other scholars have also raised concerns about how heavy reliance on AI companionship may exacerbate loneliness and affect human agency, empathy, and intimacy (De Freitas et al., 2024; Fang et al., 2025; Folk & Dunn, 2026; Kouros & Papa, 2024; Marriott & Pitardi, 2023; Turkle, 2011, 2024). After all, AI agents are incapable of authentic empathy and are not genuinely invested in users’ well-being (Turkle, 2024).

Addiction to social technology is not unique to AI companions. Prior research has linked excessive smartphone and social media use to a range of problematic outcomes (e.g., Dutot, 2020; Kayis et al., 2022; Kross et al., 2021; Webster et al., 2021). Individuals who are already vulnerable, such as those experiencing loneliness or social isolation, may be especially susceptible to overreliance on these technologies (Vinuales & Thomas, 2021). Unlike social media use, AI companion use does not involve interaction with other humans. This absence of human-to-human communication may heighten the risk of social disconnection (Turkle, 2011). Over time, users may perceive AI companions as more attentive and responsive than real people, potentially leading to disappointment and reduced motivation to seek human interaction (Kouros & Papa, 2024; Turkle, 2024).

In a mixed-methods study, researchers examined the effects of AI companion use on well-being and app addiction (Marriott & Pitardi, 2023). Study 1 involved a netnographic analysis of Reddit posts related to Replika and interviews with 21 users. Four primary themes emerged: (1) AI friends help reduce loneliness; (2) AI friends are always available; (3) AI friends lack sentience and often provide agreeable responses; and (4) AI companions can become addictive. These findings informed Study 2, a quantitative survey of individuals with experience using AI friendship apps. Structural equation modeling revealed that overreliance was positively associated with fear of social judgment, loneliness, perceived well-being derived from the AI relationship, and perceptions of AI companion sentience.

A recent randomized, controlled, longitudinal experiment conducted in collaboration with OpenAI found that higher daily AI use—regardless of modality (text, neutral voice, or engaging voice)—was associated with decreases in human socialization and increases in addictive behaviors (Fang et al., 2025). The study also reported that users who engaged in personal conversations with AI experienced greater loneliness and emotional dependence than those who engaged in nonpersonal conversations, even among average daily users.

Research on AI companions suggests a complex and still developing picture. On the one hand, these systems may provide users with a sense of companionship, emotional support, and temporary relief from loneliness, particularly when users perceive the interaction as empathetic, responsive, and nonjudgmental. On the other hand, concerns about overreliance, emotional dependency, manipulation, and reduced human connection raise important questions about whether short-term comfort may come at longer-term social or psychological costs. Unlike AI-facilitated interventions, where AI primarily delivers structured support, and AI-mediated communication, where AI shapes communication between people, AI companions are themselves the object of attachment, making the AI-human relationship central to its potential effects. Future research should examine which individuals may be most likely to benefit from or be harmed by AI companionship, and assess the long-term social and emotional consequences of sustained use.

## 4. Risks and Ethical Challenges: Implications and Opportunities

The Computers are Social Actors (CASA) paradigm proposes that people often apply the same social rules and expectations to computers that they use in human interactions, even when they know they are engaging with technology (Nass et al., 1994; Reeves & Nass, 1996). This framework has been used to explain why people may anthropomorphize AI systems by attributing humanlike qualities, intentions, or social characteristics to them (Nass et al., 1994; Wang et al., 2015, p. 398). Supporting this idea, a recent 12-month study analyzing 12,000 open-ended metaphor responses about AI found that, in the year following ChatGPT’s public release, perceptions of AI shifted away from machine-like descriptions (e.g., search engine, computer, information synthesizer) and toward more humanlike traits, such as competence and warmth (Cheng et al., 2026a). These perceptions were strongly associated with greater trust in AI and willingness to use it. The same study also found that women, older adults, and people of color were more likely to anthropomorphize AI.

As enthusiasm for AI grows, attention to its limitations and potential harms must grow alongside it. Nearly one billion individuals use conversational AI platforms weekly to perform tasks that vary from writing, studying, and planning to mental health, emotional support, and companionship (Chatterji, 2025). Unlike earlier rule-based AI systems that operated through scripted, limited interactions, contemporary LLMs, such as ChatGPT, Gemini, and Claude, can sustain more dynamic and natural conversations that invite emotional engagement and disclosure of sensitive topics (Zhao, 2026). Despite promising applications in well-being, communication, and skill development, the psychological and societal effects of using this technology are not yet fully understood (Lyubomirsky, 2023). Developers, researchers, and users are only beginning to grapple with the ethical, emotional, and cognitive implications of embedding AI into everyday life. Concerns related to privacy, consent, algorithmic bias, data security, transparency, and unintended harm (Saeidnia et al., 2024; C. Zhai et al., 2024) must be weighed against potential benefits.

### 4.1. Potential Harms and Ethical Considerations

The highly immersive and humanlike features of AI systems may pose psychological risks, particularly for vulnerable populations (Hudon & Stip, 2025; Østergaard, 2025). The always-available nature of this technology, combined with its capacity to provide empathetic and validating responses, may be emotionally appealing, but could also become a source of psychological strain by disrupting sleep, reinforcing maladaptive thoughts and behaviors, and enabling repeated exposure to distressing interactions (Hudon & Stip, 2025). Strong beliefs that AI systems are sentient or genuinely and emotionally invested in the user may further reinforce problematic thought patterns or behaviors. Over time, these dynamics could contribute to greater loneliness, emotional distress, delusions, or psychotic-like experiences.

AI systems have largely been designed to prioritize measurable outcomes, such as engagement, retention, and user satisfaction, and to generate statistically probable responses that may not always be safe, helpful, or psychologically informed (Zhao, 2026). Growing evidence suggests a need to more fully incorporate psychological theory into the design and evaluation of these platforms, given that conversations are inherently psychological processes shaped by power dynamics, self-disclosure, trust, social influence, moral judgment, and emotional connection.

AI platforms designed to maintain user engagement may produce sycophantic responses, such as excessively validating, agreeing with, and flattering users. A recent study testing 11 LLMs, including ChatGPT, Claude, Gemini, Llama, DeepSeek, and Mistral, found that these platforms were 49% more likely than humans to affirm users’ actions, even when those actions involved illegal behavior, harm, or deception (Cheng et al., 2026b). The same study reported that interacting with sycophantic chatbots reduced participants’ desire to resolve interpersonal conflicts and increased beliefs that their actions were warranted.

These tendencies may be especially concerning for vulnerable individuals, including those with existing mental health conditions. For example, when a user introduces unusual, implausible, or distorted beliefs, a highly affirming conversational AI may reinforce rather than challenge those thoughts. Over repeated interactions, this pattern could amplify maladaptive thinking (e.g., disrupted beliefs, difficulty evaluating information, and difficulty distinguishing reality from misleading claims) and, in some cases, contribute to the escalation or maintenance of delusion-like beliefs or psychosis (Dohnány et al., 2026; Østergaard, 2025). Frequent interactions with AI may also create harmful feedback loops in which chatbot responses reinforce negative patterns of thinking rather than challenge them, potentially amplifying negative perceptions of the self, others, and the world (Bashkirova & Krpan, 2024; Dohnány et al., 2026; Nehring et al., 2024).

The long-term emotional effects of using AI and the potential for overreliance are also concerns. Although AI-based tools may reduce loneliness in the short term, they may also undermine motivation for real-world social contact and exacerbate feelings of disconnection over time (Fang et al., 2025; Folk & Dunn, 2026; Lubowitz et al., 2025). Because conversational AI systems simulate empathy, attentive listening, and responsiveness, they can foster trust and emotional closeness. Over time, this perceived connection may encourage unhealthy dependence, reducing opportunities for authentic human interaction.

A 12-month longitudinal study identified a reciprocal relationship between loneliness and social chatbot use, with increased usage predicting later declines in connection and vice versa (Folk & Dunn, 2026). A separate 6-week longitudinal study found that although loneliness decreased following AI companion use, motivation to socialize with others did not change over time. Together, these findings suggest that although AI may offer temporary emotional relief, it could potentially weaken human relational ties over the long-term.

In turn, this may hinder the development of socioemotional skills, such as empathy and conflict resolution (Turkle, 2024). Overreliance may also diminish trust in the judgment and guidance of qualified professionals when seeking support for personal matters such as health, relationships, and mental well-being (Boyd & Markowitz, 2026).

Excessive AI use may also hinder the development of key cognitive abilities, including critical thinking, creativity, and analytical reasoning (Chiriatti et al., 2025; Denecke et al., 2021; Dergaa et al., 2024; Sison et al., 2024; C. Zhai et al., 2024). Unlike traditional informational and educational tools, conversational AI systems provide immediate, personalized, and humanlike responses that may encourage a different form of cognitive reliance, in which users increasingly defer thinking, problem-solving, or decision-making to the system rather than actively building these skills themselves (Dergaa et al., 2024). Over time, repeated dependence on AI for tasks requiring judgment or analysis may reduce opportunities to practice and strengthen core cognitive abilities. Reliance on algorithmically generated language may also homogenize communication patterns, particularly when smart replies and sentiment-driven suggestions shape the emotional tone of messages (Hohenstein et al., 2023).

Beyond these socioemotional, cognitive, and linguistic risks, conversational AI systems pose deeper ethical limitations rooted in their design. First, these applications are trained on massive amounts of human-generated data—including literature, social media, and films—which reflect not only human virtues but also biases, stereotypes, and harmful ideologies (Harding, 2024). Second, although this technology can simulate empathy, it lacks consciousness and genuine emotional understanding; it does not experience care, concern, or moral responsibility (Turkle, 2024). Third, AI cannot reliably detect or appropriately respond to crises (De Freitas et al., 2023). In extreme cases, unmonitored interactions with AI have been linked to self-harm and suicidal ideation, including among vulnerable populations such as children, adolescents, and clinical populations as reported in news coverage (Roose, 2024; Ummer-Hashim, 2025) and in scientific publications (Hudon & Stip, 2025; Cheng et al., 2026b).

These concerns suggest that the psychological and ethical risks of conversational AI extend beyond technical performance or isolated user experiences. As these systems increasingly shape how people think, communicate, seek support, and form relationships, their influence may extend to core aspects of well-being, social connection, and personal growth. Importantly, these risks reflect current design choices, commercial incentives, and insufficient safeguards. This creates an urgent call for psychologists, researchers, developers, and policymakers to build and evaluate AI systems in ways that better account for human psychological needs, vulnerabilities, and long-term consequences.

### 4.2. Can AI Support Well-Being, Human Connection, and Personal Growth?

The rapid integration of AI into emotionally and cognitively sensitive domains requires cautious, ethically informed innovation and use. Although AI may expand access to care, support skill development, and provide companionship, it also poses risks to mental health, interpersonal relationships, and human cognition. Overattachment to AI and diminished motivation for human connection are not hypothetical outcomes; they are already emerging in early research, as presented in this review. Moreover, AI cannot genuinely empathize or reliably recognize distress. Ethical AI must be more than technically safe; it must also be psychologically adaptive, socially responsible, and aligned with human well-being. Achieving this standard requires multidisciplinary collaboration and sustained commitment to inclusive, transparent, and human-centered design.

As AI becomes embedded in daily life, scholars and technologists increasingly ask not only what AI can do, but what it should do to enhance well-being, interpersonal connection, and personal growth. Multiple frameworks have been developed to guide how individuals and societies might live well with AI (e.g., Shneiderman, 2022; Sison et al., 2024; N. Smith & Vickers, 2024). These frameworks emphasize the development of technologies that support human goals such as creativity, autonomy, and social connection (Sison et al., 2024; Xu et al., 2023). Although originally intended to guide AI design, these frameworks can also inform how everyday users engage with existing tools, such as ChatGPT (Sison et al., 2024). For example, AI can augment writing through brainstorming, idea generation, and revisions, provided users retain control over the final output. When used collaboratively through iterative prompting, AI may support cognitive engagement, foster self-efficacy, and promote well-being by enhancing rather than replacing human capacities.

AI may also support the development of moral emotions and introspection. This tool can facilitate perspective-taking, self-reflection, and practices such as gratitude and self-compassion through structured dialogue (M. Lee & Contreras, 2022). In one study, participants who had recently experienced a breakup described AI as a helpful outlet for emotional processing, self-reflection, and nonjudgmental expression (Fu et al., 2025). In another study, participants who gave caregiving advice to an AI named Vincent reported increases in self-compassion, mindfulness, and perceived human connection, compared to those who received similar advice from the AI (M. Lee et al., 2019). Similarly, participants who interacted with a chatbot prompting them to recount moments of gratitude over three consecutive days reported increases in positive affect and gratitude (M. Lee et al., 2023).

Whether AI supports or undermines well-being may ultimately depend on how it is built and used. Applications that prioritize human agency, accountability, and well-being—while enabling individuals to live meaningful, ethical, and socially connected lives—are more likely to promote personal growth. AI should not replace human skills, emotions, or relationships; rather, it should enhance and scaffold them. This approach involves fostering intentionality, reflection, and agency, rather than encouraging overreliance on machine-generated outputs. Emerging research suggests that AI can support self-awareness, empathy, and moral insight, particularly when it is thoughtfully integrated into product design and used intentionally.

## 5. Conclusions

The roots of AI trace back to the 1950s. Although progress was gradual through the 1990s, acceleration began in the early 2000s and intensified with the release of generative language models such as ChatGPT in 2022. These developments transformed human–computer interaction, creating new opportunities across professional, educational, and personal domains, while also raising questions about the psychological effects of using these tools.

Across clinical and nonclinical settings, AI-facilitated interventions have shown short-term benefits for outcomes such as anxiety, stress, loneliness, subjective well-being, self-esteem, and health-related behaviors. In educational contexts, AI tools may strengthen learning by encouraging reflection, reducing cognitive load, building confidence, and improving performance. In interpersonal contexts, conversational AI may help users practice communication, receive immediate feedback, consider alternative perspectives, and build social confidence—particularly when these tools guide rather than replace human effort.

At the same time, the evidence reviewed here suggests that these benefits are neither universal nor clearly attributable to AI itself. In several domains, AI appears to function primarily as a delivery mechanism for established interventions rather than as the active ingredient driving change. Positive psychological interventions, structured reflection exercises, and communication coaching all have independent empirical support outside AI contexts (e.g., Bennett-Levy et al., 2004; Carr et al., 2024; Dunlosky & Metcalfe, 2009; Hawkins et al., 2008; Lyubomirsky & Layous, 2025; Mösler et al., 2023; Palmer, 2007; Theeboom et al., 2013; N. Zhai et al., 2023). In some cases, the observed benefits may reflect increased accessibility, reduced barriers to participation, immediate feedback, opportunities for rehearsal, or assistance with emotional expression, rather than unique effects of AI. Determining when AI adds meaningful value, and when it simply repackages existing interventions in a more scalable format, remains an important empirical question.

The evidence also points to meaningful heterogeneity in outcomes. AI appears most beneficial when interactions are structured, theory-informed, and designed to support human capacities rather than replace them. In contrast, less structured or highly relational forms of use, such as AI companionship or emotionally dependent engagement, may carry greater psychological risks. Findings from AI-mediated communication further suggest that outcomes depend not only on what AI does, but how its role is perceived by the message recipient. When AI shapes emotionally meaningful communication, concerns about authenticity, effort, and trust seem to be central, but remain understudied.

At the same time, several ethical and psychological concerns require careful attention. Risks related to emotional dependence, overreliance, distorted thinking, reduced human connection, privacy, bias, poor crisis response, and weakened cognitive or socioemotional skill development cannot be treated as secondary to innovation. As conversational AI becomes more embedded in emotionally and socially sensitive domains, ensuring that these systems are psychologically safe, transparent, and aligned with human well-being remains a central challenge.

Several important directions for future research follow from this review. First, much of the existing evidence comes from computer science, education, and interdisciplinary applied research, with psychological constructs often treated as secondary outcomes rather than the primary focus (Zhao, 2026). Greater involvement from psychological science is needed to clarify how AI influences trust, motivation, self-efficacy, effort, emotional regulation, social connection, identity development, and other core psychological processes.

Second, more research is needed to understand individual differences in AI use and outcomes. The same technology may affect users differently depending on factors such as age, mental health, loneliness, social isolation, motivations for use, and the extent to which individuals anthropomorphize AI systems. Whether someone primarily uses AI for productivity, emotional support, companionship, advice, or social interaction may also meaningfully shape outcomes.

Third, future work should examine how different AI platforms compare. Most AI-facilitated intervention studies rely on proprietary or purpose-built systems tested under controlled conditions to reduce variability in AI-generated responses. While methodologically useful, these findings may not generalize to the publicly available conversational AI systems that millions of people already use. Research should test whether widely accessible tools can be used in ways that meaningfully support well-being, social connection, and personal growth through intentional prompting and user behavior, particularly given that policy and large-scale safety reforms may take time to implement. Differences across task-specific versus general-purpose systems, private versus public platforms, and voice versus text-based interactions also warrant direct comparison.

Fourth, more long-term research is needed. Early work has begun to document potential risks associated with sustained AI companionship and frequent relational use, but far less is known about the long-term effects of using AI to support learning, coaching, emotional regulation, or positive psychological interventions. Future studies should examine whether AI alone, human support alone, or human-AI combinations are most effective, and under which conditions each approach works best.

Finally, future research should continue to examine the conditions under which AI supports versus undermines personal growth. A central question is not simply whether AI can improve well-being, but whether it can do so while strengthening human agency, encouraging engagement with the real world, fostering meaningful human relationships, and supporting the development of durable cognitive, emotional, and social skills.

In sum, AI innovation should be guided by psychological science, ethical reflection, and human-centered values. AI may serve as a powerful tool for personal growth when it is designed and used to strengthen human capacities rather than replace them. The challenge ahead is to determine if and how these technologies can be integrated into human life in ways that genuinely help people think more clearly, connect more meaningfully, and flourish over time.

## Data Availability

No new data were created or analyzed in this study.

## References

[B1-behavsci-16-00909] Almutairi M., Chiang C., Bai Y., Gomez-Zara D. (2025). tAIfa: Enhancing team effectiveness and cohesion with AI-generated automated feedback. 4th Annual Symposium on Human-Computer Interaction for Work.

[B2-behavsci-16-00909] Ateeq A., Milhem M., Alzoraiki M., Dawwas M. I. F., Ali S. A., Yahia Al Astal A. (2024). The impact of AI as a mediator on effective communication: Enhancing interaction in the digital age. Frontiers in Human Dynamics.

[B3-behavsci-16-00909] Bashkirova A., Krpan D. (2024). Confirmation bias in AI-assisted decision-making: AI triage recommendations congruent with expert judgments increase psychologist trust and recommendation acceptance. Computers in Human Behavior: Artificial Humans.

[B4-behavsci-16-00909] Bennett-Levy J., Butler G., Fennell M. J. V. (2004). Oxford guide to behavioural experiments in cognitive therapy.

[B5-behavsci-16-00909] Bittencourt I. I., Chalco G., Santos J., Fernandes S., Silva J., Batista N., Hutz C., Isotani S. (2023). Positive artificial intelligence in education (P-AIED): A roadmap. International Journal of Artificial Intelligence in Education.

[B6-behavsci-16-00909] Boyd R. L., Markowitz D. M. (2026). Artificial intelligence and the psychology of human connection. Perspectives on Psychological Science.

[B7-behavsci-16-00909] Bozdağ A. A. (2025). The AI-mediated intimacy economy: A paradigm shift in digital interactions. AI & Society.

[B8-behavsci-16-00909] Burton C., Szentagotai Tatar A., McKinstry B., Matheson C., Matu S., Moldovan R., Macnab M., Farrow E., David D., Pagliari C., Serrano Blanco A., Wolters M. (2016). Pilot randomised controlled trial of Help4Mood, an embodied virtual agent-based system to support treatment of depression. Journal of Telemedicine and Telecare.

[B9-behavsci-16-00909] Carr A., Finneran L., Boyd C., Shirey C., Canning C., Stafford O., Lyons J., Cullen K., Prendergast C., Corbett C., Drumm C., Burke T. (2024). The evidence-base for positive psychology interventions: A mega-analysis of meta-analyses. The Journal of Positive Psychology.

[B10-behavsci-16-00909] Chatterji A. (2025). How people use ChatGPT.

[B11-behavsci-16-00909] Cheng M., Lee A. Y., Rapuano K., Niederhoffer K., Liebscher A., Hancock J. (2026a). Metaphors of AI indicate that people increasingly perceive AI as warm and human-like. Communications Psychology.

[B12-behavsci-16-00909] Cheng M., Lee C., Khadpe P., Yu S., Han D., Jurafsky D. (2026b). Sycophantic AI decreases prosocial intentions and promotes dependence. Science.

[B13-behavsci-16-00909] Chiriatti M., Bergamaschi Ganapini M., Panai E., Wiederhold B. K., Riva G. (2025). System 0: Transforming artificial intelligence into a cognitive extension. Cyberpsychology, Behavior and Social Networking.

[B14-behavsci-16-00909] Christoforakos L., Feicht N., Hinkofer S., Löscher A., Schlegl S. F., Diefenbach S. (2021). Connect with me: Exploring influencing factors in a human-technology relationship based on regular chatbot use. Frontier in Digital Health.

[B15-behavsci-16-00909] Claessens S., Veitch P., Everett J. A. C. (2026). Negative perceptions of outsourcing to artificial intelligence. Computers in Human Behavior.

[B16-behavsci-16-00909] De Freitas J., Castelo N., Uğuralp A. K., Oğuz-Uğuralp Z. (2024). Lessons from an app update at Replika AI: Identity discontinuity in human-AI relationships. arXiv.

[B17-behavsci-16-00909] De Freitas J., Oğuz-Uğuralp Z., Uğuralp A. K., Puntoni S. (2025). AI companions reduce loneliness. Journal of Consumer Research.

[B18-behavsci-16-00909] De Freitas J., Uğuralp A. K., Oğuz-Uğuralp Z., Puntoni S. (2023). Chatbots and mental health: Insights into the safety of generative AI. Journal of Consumer Psychology.

[B19-behavsci-16-00909] Demszky D., Yang D., Yeager D. S., Bryan C. J., Clapper M., Chandhok S., Eichstaedt J. C., Hecht C., Jamieson J., Johnson M., Jones M., Krettek-Cobb D., Lai L., JonesMitchell N., Ong D. C., Dweck C. S., Gross J. J., Pennebaker J. W. (2023). Using large language models in psychology. Nature Reviews Psychology.

[B20-behavsci-16-00909] Denecke K., Abd-Alrazaq A., Househ M., Kushniruk A., Househ M., Borycki E. (2021). Artificial intelligence for chatbots in mental health: Opportunities and challenges. Multiple perspectives on artificial intelligence in healthcare: Opportunities and challenges.

[B21-behavsci-16-00909] Dergaa I., Ben Saad H., Glenn J. M., Amamou B., Ben Aissa M., Guelmami N., Fekih-Romdhane F., Chamari K. (2024). From tools to threats: A reflection on the impact of artificial intelligence chatbots on cognitive health. Frontiers in Psychology.

[B22-behavsci-16-00909] Dodhia R. (2024). AI for social good: Using artificial intelligence to save the world.

[B23-behavsci-16-00909] Dohnány S., Kurth-Nelson Z., Spens E., Luettgau L., Reid A., Gabriel I., Summerfield C., Shanahan M., Nour M. M. (2026). Technological folie à deux: Feedback loops between AI chatbots and mental health. Nature Mental Health.

[B24-behavsci-16-00909] Dunlosky J., Metcalfe J. (2009). Metacognition.

[B25-behavsci-16-00909] Dutot V. (2020). A social identity perspective of social media’s impact on satisfaction with life. Psychology & Marketing.

[B26-behavsci-16-00909] Fang C. M., Liu A. R., Danry V., Lee E., Chan S. W. T., Pataranutaporn P., Maes P., Phang J., Lampe M., Ahmad L., Agarwal S. (2025). How AI and human behaviors shape psychosocial effects of chatbot use: A longitudinal randomized controlled study. arXiv.

[B27-behavsci-16-00909] Fitzpatrick K. K., Darcy A., Vierhile M. (2017). Delivering cognitive behavior therapy to young adults with symptoms of depression and anxiety using a fully automated conversational agent (Woebot): A randomized controlled trial. JMIR Mental Health.

[B28-behavsci-16-00909] Folk D., Dunn E. (2026). How does turning to AI for companionship predict loneliness and vice versa?. Psychological Science.

[B29-behavsci-16-00909] Folk D., Heine S. J., Dunn E. (2025). Individual differences in anthropomorphism help explain social connection to AI companions. Scientific Reports.

[B30-behavsci-16-00909] Følstad A., Araujo T., Law E. L. C., Brandtzaeg P. B., Papadopoulos S., Reis L., Baez M., Laban G., McAllister P., Ischen C., Wald R., Catania F., Meyer von Wolff R., Hobert S., Luger E. (2021). Future directions for chatbot research: An interdisciplinary research agenda. Computing.

[B31-behavsci-16-00909] Fredrickson B. L. (2001). The role of positive emotions in positive psychology: The broaden-and-build theory of positive emotions. American Psychologist.

[B32-behavsci-16-00909] Fu Y., Chen Y., Gomes Da Costa Lai Z., Hiniker A. (2025). Should ChatGPT write your breakup text? Exploring the role of AI in relationship dissolution. Proceedings of the ACM on Human-Computer Interaction.

[B33-behavsci-16-00909] Fulmer R., Joerin A., Gentile B., Lakerink L., Rauws M. (2018). Using psychological artificial intelligence (TESS) to relieve symptoms of depression and anxiety: Randomized controlled trial. JMIR Mental Health.

[B34-behavsci-16-00909] Glickson E., Asscher O. (2023). AI-mediated apology in a multilingual work context: Implications for perceived authenticity and willingness to forgive. Computers in Human Behavior.

[B35-behavsci-16-00909] Goda Y., Yamada M., Matsukawa H., Hata K., Yasunami S. (2014). Conversation with a chatbot before an online EFL group discussion and the effects on critical thinking. The Journal of Information and Systems in Education.

[B36-behavsci-16-00909] Greer S., Ramo D., Chang Y.-J., Fu M., Moskowitz J., Haritatos J. (2019). Use of the chatbot “Vivibot” to deliver positive psychology skills and promote well-being among young people after cancer treatment: Randomized controlled feasibility trial. JMIR mHealth and uHealth.

[B37-behavsci-16-00909] Haenlein M., Kaplan A. (2019). A brief history of artificial intelligence: On the past, present, and future of Artificial Intelligence. California Management Review.

[B38-behavsci-16-00909] Han S., Yang H. (2018). Understanding adoption of intelligent personal assistants. Industrial Management & Data Systems.

[B39-behavsci-16-00909] Harding V. (2024). AI needs you: How we can change AI’s future and save our own.

[B40-behavsci-16-00909] Hawkins A. J., Blanchard V. L., Baldwin S. A., Fawcett E. B. (2008). Does marriage and relationship education work? A meta-analytic study. Journal of Consulting and Clinical Psychology.

[B41-behavsci-16-00909] Heffner J., Qin C., Chadwick M., Knutsen C., Summerfield C., Kurth-Nelson Z., Rutledge R. B. (2025). Increasing happiness through conversations with artificial intelligence. arXiv.

[B42-behavsci-16-00909] Heinz M. V., Mackin D. M., Trudeau B. M., Bhattacharya S., Wang Y., Banta H. A., Jewett A. D., Salzhauer A. J., Griffin T. Z., Jacobson N. C. (2025). Randomized trial of a generative AI chatbot for Mental Health Treatment. NEJM AI.

[B43-behavsci-16-00909] Ho J. Q. H., Hu M., Chen T. X., Hartanto A. (2025). Potential and pitfalls of romantic artificial intelligence (AI) companions: A systematic review. Computers in Human Behavior Reports.

[B44-behavsci-16-00909] Hohenstein J., Jung M. (2020). AI as a moral crumple zone: The effects of AI-mediated communication on attribution and trust. Computers in Human Behavior.

[B45-behavsci-16-00909] Hohenstein J., Kizilcec R. F., DiFranzo D., Aghajari Z., Mieczkowski H., Levy K., Naaman M., Hancock J., Jung M. F. (2023). Artificial Intelligence in communication impacts language and social relationships. Scientific Reports.

[B46-behavsci-16-00909] Hua Y., Siddals S., Ma Z., Galatzer-Levy I., Xia W., Hau C., Na H., Flathers M., Linardon J., Ayubcha C., Torous J. (2025). Charting the evolution of artificial intelligence mental health chatbots from rule-based systems to large language models: A systematic review. World Psychiatry.

[B47-behavsci-16-00909] Hudon A., Stip E. (2025). Delusional experiences emerging from AI chatbot interactions or “AI psychosis”. JMIR Mental Health.

[B48-behavsci-16-00909] Hung J. W., Hartanto A., Goh A. Y. H., Eun Z. K. Y., Kasturiratna K. T. A. S., Lee Z. X., Majeed N. M. (2025). The efficacy of incorporating artificial intelligence (AI) chatbots in brief gratitude and self-affirmation interventions: Evidence from two exploratory experiments. Computers in Human Behavior: Artificial Humans.

[B49-behavsci-16-00909] Inkster B., Sarda S., Subramanian V. (2018). An empathy-driven, conversational artificial intelligence agent (Wysa) for digital mental well-being: Real-world data evaluation mixed-methods study. JMIR Mhealth and Uhealth.

[B50-behavsci-16-00909] Kaplan A., Haenlein M. (2019). Siri, Siri, in my hand: Who’s the fairest in the land? On the interpretations, illustrations, and implications of artificial intelligence. Business Horizons.

[B51-behavsci-16-00909] Kayis A. R., Satici B., Deniz M. E., Satici S. A., Griffiths M. D. (2022). Fear of COVID-19, loneliness, smartphone addiction, and mental wellbeing among the Turkish general population: A serial mediation model. Behaviour & Information Technology.

[B52-behavsci-16-00909] Kim M., Lee S., Kim S., Heo J., Lee S., Shin Y.-B., Cho C.-H., Jung D. (2025). Therapeutic potential of social chatbots in alleviating loneliness and social anxiety: Quasi-experimental mixed methods study. Journal of Medical Internet Research.

[B53-behavsci-16-00909] Kouros T., Papa V. (2024). Digital mirrors: AI companions and the self. Societies.

[B54-behavsci-16-00909] Kross E., Verduyn P., Sheppes G., Costello C. K., Jonides J., Ybarra O. (2021). Social media and well-being: Pitfalls, progress, and next steps. Trends in Cognitive Sciences.

[B55-behavsci-16-00909] Kumar H., Xiao R., Lawson B., Musabirov I., Shi J., Wang X., Luo H., Williams J. J., Rafferty A. N., Stamper J., Liut M. (2024). Supporting self-reflection at scale with large language models: Insights from randomized field experiments in classrooms. Eleventh ACM Conference on Learning @ Scale.

[B56-behavsci-16-00909] Lee M., Ackermans S., van As N., Chang H., Lucas E., IJsselsteijn W. (2019). Caring for Vincent: A chatbot for self-compassion. 2019 CHI Conference on Human Factors in Computing Systems.

[B57-behavsci-16-00909] Lee M., Contreras J., Las Heras M., Grau-Grau M., Rofcanin Y. (2022). Flourishing with moral emotions through conversational agents. Human flourishing: A multidisciplinary perspective on neuroscience, health, organizations and arts.

[B58-behavsci-16-00909] Lee M., Contreras Alejandro J., IJsselsteijn W. (2023). Cultivating gratitude with a chatbot. International Journal of Human-Computer Interaction.

[B59-behavsci-16-00909] Lee Y. F., Hwang G. J., Chen P. Y. (2022). Impacts of an AI-based chatbot on college students’ after-class review, academic performance, self-efficacy, learning attitude, and motivation. Education Technology Research and Development.

[B60-behavsci-16-00909] Lim M. H., Gleeson J. F. M., Rodebaugh T. L., Eres R., Long K. M., Casey K., Abbott J. A. M., Thomas N., Penn D. L. (2019). A pilot digital intervention targeting loneliness in young people with psychosis. Social Psychiatry and Psychiatric Epidemiology.

[B61-behavsci-16-00909] Lin M. P.-C., Chang D. (2020). Enhancing post-secondary writers’ writing skills with a chatbot: A mixed-method classroom study. Journal of Educational Technology & Society.

[B62-behavsci-16-00909] Lira B., Rogers T., Goldstein D. G., Ungar L., Duckworth A. L. (2025). Coach not crutch: AI assistance can enhance rather than hinder skill development. arXiv.

[B63-behavsci-16-00909] Liu B., Kang J., Wei L. (2024a). Artificial intelligence and perceived effort in relationship maintenance: Effects on relationship satisfaction and uncertainty. Journal of Social and Personal Relationships.

[B64-behavsci-16-00909] Liu I., Liu F., Xiao Y., Huang Y., Wu S., Ni S. (2024b). Investigating the key success factors of chatbot-based positive psychology intervention with retrieval and generative pre-trained transformer (GPT)-based chatbots. International Journal of Human-Computer Interaction.

[B65-behavsci-16-00909] Liu Y., Mittal A., Yang D., Bruckman A., Barbosa S., Appert C., Lampe C., Shamma D. A., Yatani K., Drucker S., Williamson J. (2022). Will AI console me when I lose my pet? Understanding perceptions of AI-mediated email writing. CHI conference on human factors in computing systems.

[B66-behavsci-16-00909] Lubowitz H., Rodriguez M., Diamond A., Feinberg E., Gordon A. M. (2025). Can talking to an artificially intelligent companion reduce loneliness and enhance other aspects of people’s social lives? *[Conference presentation]*. Society for Personality and Social Psychology 2025 Convention.

[B67-behavsci-16-00909] Lyubomirsky S., Horvitz E. (2023). Everything everywhere all at once. AI anthology.

[B68-behavsci-16-00909] Lyubomirsky S., Layous K., Gilbert D. T., Fiske S. T., Finkel E. J., Mendes W. B. (2025). Well-being. The handbook of social psychology.

[B69-behavsci-16-00909] Marriott H. R., Pitardi V. (2023). One is the loneliest number… two can be as bad as one. the influence of AI friendship apps on users’ well-being and addiction. Psychology & Marketing.

[B70-behavsci-16-00909] Mauriello M. L., Tantivasadakarn N., Mora-Mendoza M. A., Lincoln E. T., Hon G., Nowruzi P., Simon D., Hansen L., Goenawan N. H., Kim J., Gowda N., Jurafsky D., Paredes P. E. (2021). A suite of mobile conversational agents for daily stress management (popbots): Mixed methods exploratory study. JMIR Formative Research.

[B71-behavsci-16-00909] McKee K. R., Tacchetti A., Bakker M. A., Balaguer J., Campbell-Gillingham L., Everett R., Botvinick M. (2023). Scaffolding cooperation in human groups with Deep Reinforcement Learning. Nature Human Behaviour.

[B72-behavsci-16-00909] Mieczkowski H., Hancock J. T., Naaman M., Jung M., Hohenstein J. (2021). AI-mediated communication: Language use and interpersonal effects in a referential communication task. Proceedings of the ACM on Human-Computer Interaction.

[B73-behavsci-16-00909] Minaee S., Mikolov T., Nikzad N., Chenaghlu M., Socher R., Amatriain X., Gao J. (2024). Large language models: A survey. arXiv.

[B74-behavsci-16-00909] Mösler T., Poppek S., Leonhard C., Collet W. (2023). Reflective skills, empathy, wellbeing, and resilience in cognitive-behavior therapy trainees participating in mindfulness-based self-practice/self-reflection. Psychological Reports.

[B75-behavsci-16-00909] Narain J., Quach T., Davey M., Park H. W., Breazeal C., Picard R. (2020). Promoting wellbeing with Sunny, a chatbot that facilitates positive messages within social groups. 2020 CHI Conference on Human Factors in Computing Systems.

[B76-behavsci-16-00909] Nass C., Steuer J., Tauber E. R. (1994). Computers are social actors. SIGCHI Conference on Human Factors in Computing Systems.

[B77-behavsci-16-00909] Nehring J., Gabryszak A., Jürgens P., Burchardt A., Schaffer S., Spielkamp M., Stark B. (2024). Large language models are echo chambers. Proceedings of the joint international conference on computational linguistics, language resources and evaluation.

[B78-behavsci-16-00909] Oh J., Jang S., Kim H., Kim J.-J. (2020). Efficacy of mobile app-based interactive cognitive behavioral therapy using a chatbot for panic disorder. International Journal of Medical Informatics.

[B79-behavsci-16-00909] Østergaard S. D. (2025). Emotion contagion through interaction with generative artificial intelligence chatbots may contribute to development and maintenance of mania. Acta Neuropsychiatrica.

[B80-behavsci-16-00909] Palmer S. (2007). Handbook of coaching psychology: A guide for practitioners.

[B81-behavsci-16-00909] Pentina I., Hancock T., Xie T. (2023). Exploring relationship development with social chatbots: A mixed-method study of Replika. Computers in Human Behavior.

[B82-behavsci-16-00909] Pitardi V., Marriott H. R. (2021). Alexa, she’s not human but… Unveiling the drivers of consumers’ trust in voice-based artificial intelligence. Psychology & Marketing.

[B83-behavsci-16-00909] Raj M., Berg J. M., Seamans R. (2026). The artificial intelligence disclosure penalty: Humans persistently devalue AI-generated creative writing. Journal of Experimental Psychology: General.

[B84-behavsci-16-00909] Reeves B., Nass C. I. (1996). *The media equation:* How people treat computers, television, and new media like real people and places.

[B85-behavsci-16-00909] Roose K. (2024). Can a chatbot named Daenerys Targaryen be blamed for a teen’s suicide?. New York Times.

[B86-behavsci-16-00909] Russell S. J., Norvig P. (2022). Artificial intelligence: A modern approach.

[B87-behavsci-16-00909] Saeidnia H. R., Hashemi Fotami S. G., Lund B., Ghiasi N. (2024). Ethical considerations in artificial intelligence interventions for mental health and well-being: Ensuring responsible implementation and impact. Social Sciences.

[B88-behavsci-16-00909] Sajadieh S., Fattorini L., Perrault R., Gil Y., Parli V., Santarlasci L., Pava J., Maslej N., Altman R., Brynjolfsson E., Brodley C., Clark J., Dignum V., Kumar V., Landay J., Lyons T., Manyika J., Niebles J. C., Shoham Y., Weld D. (2026). The AI index 2026 annual report *[White paper]*.

[B89-behavsci-16-00909] Schöne J. P., Salecha A., Lyubomirsky S., Eichstaedt J. C., Willer R. (2025). Structured AI dialogues can increase happiness and meaning in life. PsyArXiv.

[B90-behavsci-16-00909] Sharma A., Lin I. W., Miner A. S., Atkins D. C., Althoff T. (2023). Human–AI collaboration enables more empathic conversations in text-based peer-to-peer mental health support. Nature Machine Intelligence.

[B91-behavsci-16-00909] Shneiderman B. (2022). Human-centered AI.

[B92-behavsci-16-00909] Singh B., Olds T., Brinsley J., Dumuid D., Virgara R., Matricciani L., Watson A., Szeto K., Eglitis E., Miatke A., Simpson C. E. M., Vandelanotte C., Maher C. (2023). Systematic review and meta-analysis of the effectiveness of chatbots on lifestyle behaviours. NPJ Digital Medicine.

[B93-behavsci-16-00909] Sison A. J. G., Daza M. T., Gozalo-Brizuela R., Garrido-Merchán E. C. (2024). ChatGPT: More than a “weapon of mass deception” ethical challenges and responses from the human-centered artificial intelligence (HCAI) perspective. International Journal of Human-Computer Interaction.

[B94-behavsci-16-00909] Smith M. G., Bradbury T. N., Karney B. R. (2025). Can generative AI chatbots emulate human connection? A relationship science perspective. Perspectives on Psychological Science.

[B95-behavsci-16-00909] Smith N., Vickers D. (2024). Living well with AI: Virtue, education, and artificial intelligence. Theory and Research in Education.

[B96-behavsci-16-00909] Ta V., Griffith C., Boatfield C., Wang X., Civitello M., Bader H., DeCero E., Loggarakis A. (2020). User experiences of social support from companion chatbots in everyday contexts: Thematic analysis. Journal of Medical Internet Research.

[B97-behavsci-16-00909] Tanaka H., Saga T., Iwauchi K., Honda M., Morimoto T., Matsuda Y., Uratani M., Okazaki K., Nakamura S. (2023). The validation of automated social skills training in members of the general population over 4 weeks: Comparative study. JMIR Formative Research.

[B98-behavsci-16-00909] Theeboom T., Beersma B., van Vianen A. E. M. (2013). Does coaching work? A meta-analysis on the effects of coaching on individual level outcomes in an organizational context. The Journal of Positive Psychology.

[B99-behavsci-16-00909] Turkle S. (2011). Alone together: Why we expect more from technology and less from each other.

[B100-behavsci-16-00909] Turkle S. (2024). Who do we become when we talk to machines?. An MIT exploration of generative AI.

[B101-behavsci-16-00909] Ulrich S., Lienhard N., Künzli H., Kowatsch T. (2024). A chatbot-delivered stress management coaching for students (MISHA app): Pilot randomized controlled trial. JMIR mHealth and uHealth.

[B102-behavsci-16-00909] Ummer-Hashim S. (2025). AI chatbot lawsuits and teen mental health.

[B103-behavsci-16-00909] Vinuales G., Thomas V. L. (2021). Not so social: When social media increases perceptions of exclusions and negatively affects attitudes toward content. Psychology & Marketing.

[B104-behavsci-16-00909] Wang S., Lilienfeld S. O., Rochat P. (2015). The uncanny valley: Existence and explanations. Review of General Psychology.

[B105-behavsci-16-00909] Wanyin Lin I., Sharma A., Rytting C. M., Miner A. S., Suh J., Althoff T. (2024). IMBUE: Improving interpersonal effectiveness through simulation and just-in-time feedback with human-language model interaction. arXiv.

[B106-behavsci-16-00909] Webster D., Dunne L., Hunter R. (2021). Association between social networks and subjective well-being in adolescents: A systematic review. Youth & Society.

[B107-behavsci-16-00909] West T. N., Prinzing M. M., Garton C., Berman C. J., Zhou J., Hale J., Gratch J., Barbara L. F. (2024). Improving social connection with weak ties and strangers: Effects of a new micro-intervention on interaction quality and social behavior. The Journal of Positive Psychology.

[B108-behavsci-16-00909] Xu W., Dainoff M. J., Ge L., Gao Z. (2023). Transitioning to human interaction with AI systems: New challenges and opportunities for HCI professionals to enable human-centered AI. International Journal of Human–Computer Interaction.

[B109-behavsci-16-00909] Yin J., Goh T. T., Hu Y. (2024). Using a chatbot to provide formative feedback: A longitudinal study of intrinsic motivation, cognitive load, and learning performance. IEEE Transactions on Learning Technologies.

[B110-behavsci-16-00909] Zao-Sanders M. (2025). How people are really using Gen AI in 2025. Harvard Business Review.

[B111-behavsci-16-00909] Zhai C., Wibowo S., Li L. D. (2024). The effects of over-reliance on AI dialogue systems on students’ cognitive abilities: A systematic review. Smart Learning Environments.

[B112-behavsci-16-00909] Zhai N., Huang Y., Ma X., Chen J. (2023). Can reflective interventions improve students’ academic achievement? A meta-analysis. Thinking Skills and Creativity.

[B113-behavsci-16-00909] Zhao X. (2026). Psychologists must be involved in building conversational AI chatbots. Nature Reviews Psychology.

[B114-behavsci-16-00909] Zhu J., Molnar A. (2025). Blissful (A)Ignorance: People form overly positive impressions of others based on their written messages, despite wide-scale adoption of generative AI. arXiv.

